# Failure of Engineering Artifacts: A Life Cycle Approach

**DOI:** 10.1007/s11948-012-9360-0

**Published:** 2012-03-03

**Authors:** Luca Del Frate

**Affiliations:** Faculty of Technology, Policy and Management, Delft University of Technology, Jaffalaan 5, 2628 BX Delft, The Netherlands

**Keywords:** Failure, Life cycle, Failure analysis, Product development, Failure trajectory, Sustainability

## Abstract

Failure is a central notion both in ethics of engineering and in engineering practice. Engineers devote considerable resources to assure their products will not fail and considerable progress has been made in the development of tools and methods for understanding and avoiding failure. Engineering ethics, on the other hand, is concerned with the moral and social aspects related to the causes and consequences of technological failures. But what is meant by failure, and what does it mean that a failure has occurred? The subject of this paper is how engineers use and define this notion. Although a traditional definition of failure can be identified that is shared by a large part of the engineering community, the literature shows that engineers are willing to consider as failures also events and circumstance that are at odds with this traditional definition. These cases violate one or more of three assumptions made by the traditional approach to failure. An alternative approach, inspired by the notion of product life cycle, is proposed which dispenses with these assumptions. Besides being able to address the traditional cases of failure, it can deal successfully with the problematic cases. The adoption of a life cycle perspective allows the introduction of a clearer notion of failure and allows a classification of failure phenomena that takes into account the roles of stakeholders involved in the various stages of a product life cycle.

## Introduction

Failure is a central notion both in ethics of engineering and in engineering practice. Apart from the most innocuous events which result in minor annoyances, failure of engineering artifacts raise a host of ethical questions related to allocation of responsibility, foreseeability of risks, prioritization of safety, and so on. The possibility that failure of engineered artifacts cannot be ruled out with absolute certainty is a source of concern about the introduction of any new technology.

On the engineering side, failures are at the same time *unwanted outcomes* that should be fought with the best resources provided by engineering knowledge, and one of the *main sources* of that same knowledge (Petroski 1985, 2006). In fact, the investigation of failures has played a crucial role in increasing the reliability and safety of aviation technology (Wood and Sweginnis 2006). The lessons learned from the crashes of two Comet jets in 1954, for instance, were a milestone in the understanding of metal fatigue and how to improve the design of aircraft mainframes (Wanhill 2003). Similarly to aviation, other industries and engineering specializations have achieved substantial progress by understanding the reasons behind failure events (Schlager 1994). In fact, nowadays, the systematic analysis of potential failures has become an integral part of the design process by means of tools like computer simulations (Collins 1993), Failure Modes and Effects Analysis (Stamatis 1995), Fault Tree Analysis (Xing and Amari 2008).

Moreover, the scope of the concept of failure has expanded beyond the intuitive idea of rupture and structural collapse. In aeronautics, for instance, the focus of the investigations has expanded beyond the mere “technical factors”, which were prevalent in the early days of aviation, to address safety concerns related to “human factors” and to “organizational factors” (ICAO 2009). Concurrently, engineers put an effort in taking distance from the derogatory aspects of the concept of failure and to devise a more neutral meaning. Investigative agencies like the Dutch Safety Board or the Australian Transport Safety Bureau, for instance, make clear that it is no part of their “remit to try to establish the blame, responsibility or liability attaching to any party” (The Dutch Safety Board, n.d.). Instead, their mission is to understand the causes of failures and accidents in order to prevent reoccurrences in the future.

It has to be noted, however, that better knowledge of failure, of the physical as well as of the organizational factors behind it, and increased awareness of the wider implications of failures, has not been mirrored by the creation of a unified conceptual framework shared among engineering disciplines. The urgency of finding effective measures to address failures and the complexity of failure phenomena have led to a situation of conceptual and terminological fragmentation, mostly along disciplinary divisions. Separate disciplines tend to emphasize specific aspects and to formulate definitions tailored to particular applications. As a result, although failure is a pervasive theme in engineering and despite its importance, many different uses and definitions of the concept of failure can be found in the engineering literature (Del Frate et al. 2011; Prasad et al. 1996; Tam and Gordon 2009).

The tendency towards differentiation has been somewhat balanced by unification attempts pursued by professional organizations and standardization authorities. Thanks to these efforts, a consensus, albeit partial, has coalesced around the terminology published in 1990 by the International Electrotechnical Commission (IEC) and subsequently adopted by a number of international standards. In fact, many engineering textbooks and papers dealing, one way or another, with failure include a quotation of the IEC definition:

Failure: the termination of the ability of an item to perform a required function.

This definition can be regarded as “the traditional definition of failure” (Blache and Shrivastava 1994).

Despite its intuitive appeal and the ability to capture correctly a wide range of events, it can be shown that engineers are willing to describe as failures circumstances that do not fit this traditional definition. These problematic cases suggest that some of the basic assumptions behind the traditional approach may be unwarranted. The aim of this paper, then, is to perform a detailed analysis of the traditional approach and to explore the possibility of devising a broader notion which is able to deal with the problematic cases. In doing so, this paper aims to give ethics of engineering a more accurate and present-day understanding of failure in engineering, in support of its analysis of responsibility, liability and risks.

More specifically, it is argued that a notion of failure informed by the notion of product life cycle is a suitable answer to this quest. It is shown that it is compatible with the set of failure events constituting the basis of the traditional approach; furthermore it can account for those events and circumstances that, although in violation of the traditional assumptions, engineers are willing to consider as instances of failure. The adoption of a life cycle approach allows the introduction of a clearer notion of failure and allows a rich classification of failure phenomena that takes into account the roles of various stakeholders involved in the different stages of a product life cycle.

The paper is organized as follows: ‘The “Traditional Approach” on Failure’ starts out clarifying the basic terminology related to the traditional definition. In “Four Basic Assumptions of the Traditional Approach”, the definition is analyzed in terms of four main assumptions, and then “Beyond the Traditional Approach” shows how three of these assumptions may be violated in engineering practice. In “From One Customer to Many Stakeholders”, it is argued that the traditional approach depends on a specific interpretation of the mission of product development and that this interpretation has been challenged by recent integrated models which imply the notion of product life cycle. “A New Definition of Failure” introduces the definition of *product failure* and shows that it is compatible with a life cycle approach. The new approach and its implications are analyzed in “The Life Cycle Approach in Action”. Finally, “Conclusion” summarizes and concludes the paper.

## The “Traditional Approach” on Failure

Several different definitions and characterizations of failure are available in the engineering literature (Del Frate et al. 2011; Prasad et al. 1996; Tam and Gordon 2009). In a paper on failure terminology for plant asset management, Tam and Gordon (2009) notice that the “looseness of the terminology and often overlapping shades of meaning lead to ambiguity and confusion”. Prasad et al. (1996) raise the same concern from the point of view of dependable computing where the “terminology […] is used non-uniformly by many authors and standards”.

As a paradigmatic example of this lack of uniformity let us compare two influential sources: Chapter 191 *Dependability and quality of service* of the *International Electrotechnical Vocabulary* (IEC 50(191), 1990) published by the International Electrotechnical Commission, and the *IEEE Standard Glossary of Software Engineering Terminology* (IEEE 1990) published by the Institute of Electrical and Electronics Engineers. The IEC vocabulary distinguishes between *failure* and *fault* which are defined as follows:

Failure: the termination of the ability of an item to perform a required function.

Fault: the state of an item characterized by inability to perform a required function, excluding the inability during preventive maintenance or other planned actions, or due to lack of external resources.

**Fig. 1:**
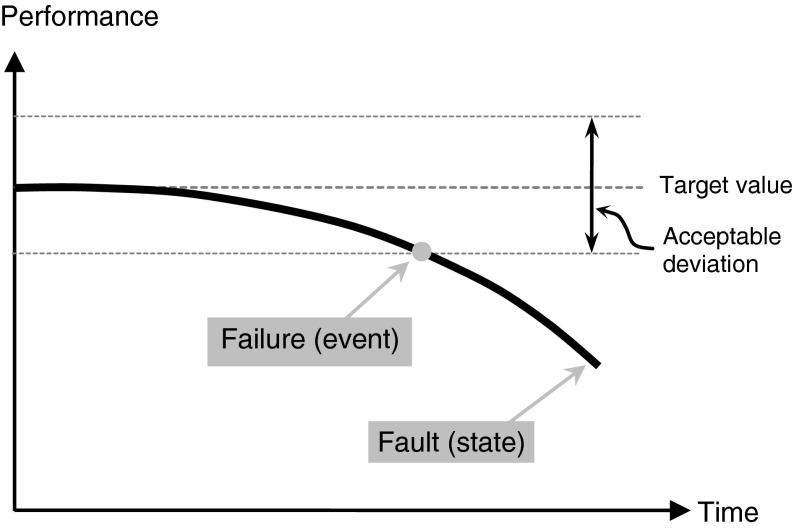
Graphical representation of the notions of *failure* and *fault* according to the IEC vocabulary. Redrawn from (Rausand and Øien 1996)

In a paper on the basic concepts of failure analysis, Rausand and Øien (1996) include a section in which they discuss the IEC terminology and clarify the distinction between failure and fault by means of the diagram that is reproduced in Fig. 1.

The curve plots the observed level of a performance variable of an item against time. Initially, the observed performance conforms to the target value and then starts to gradually deviate from it. Failure is defined as the event in which the observed performance trespasses the acceptable limits, after which the item is said to be in a fault state. The figure makes clear that the term ‘termination’ in the IEC definition should not be interpreted as total lack of ability to perform, but as the trespassing of the acceptability levels. Indeed, after a failure event an item can still be capable of performing the required function, albeit at a disappointing level.

Of course, the fact that an item under investigation has failed according to a selected performance parameter does not mean it has failed with respect to all its functions. An item can still be able to perform its main required function although the ability to perform a minor function has dropped below the acceptable limits. These are the situations that the IEC vocabulary describes as “minor failures” and are classified among the “partial failures”. In case the affected functions are considered of major importance, then the partial failure is considered of to be a “major” one; finally, at the end of the spectrum are “complete” failures which consist in the “complete inability to perform all required functions”.

It is interesting to note that the IEC vocabulary does not make provision for gradations of fault. This means that for a given item and a given function, the notion of fault is binary: either the item performs that specific (minor or major) function within the limits or it does not. Correspondingly, there are no gradations for the “ability to perform” either. So, it does not matter how close an item is to trespassing the threshold of acceptable performance for, as far as performance is within the acceptable limits, the item is described as being in a functioning state. Instead, according to the IEC vocabulary, the notion of “gradual failure” is meant to describe the process leading up to the failure event. If the discrepancy between the observed performance and the target level builds up gradually (e.g., because the item is progressively wearing out), then the ensuing event is termed a “gradual failure”; while a sudden drop beyond the acceptable limits qualifies as a “sudden failure”.

The same holds also for items performing multiple functions. Consider an item whose performance parameters are all approaching the acceptable limits but have not trespassed them yet. Then, although the overall performance is degraded, the item is still considered to be in a functioning state. The fault state will take over only when at least one of the performance parameters will trespass the boundary, at which point a partial failure will be said to have occurred. In case the item will not be removed from service and refurbished, more partial failures are likely to follow until the item will reach a state of complete failure. Therefore, in the IEC terminology the term “fault” is attributed a meaning narrower than the one prevailing in common parlance, where it can refer to defects, imperfections, or flaws that deter from the quality or value of a product which, nevertheless, may still be able to perform its required function. On the contrary, the IEC vocabulary stipulates a necessary link between the notions of fault and of lack of ability to perform one or more required functions. The discrepancies between target level and actual performance can be substantial and may affect multiple functionalities simultaneously, yet failure occurs only when at least one performance parameter has exceeded the acceptable limits.

Let us now move to the IEEE glossary (1990) which also makes a distinction between the notions of failure and fault, albeit its definitions are slightly different from the definitions given in the IEC vocabulary. The two definitions read as follows:

Failure: the inability of a system or component to perform its required functions within specified performance requirements.

Fault: (1) A defect in a hardware device or component; for example, a short circuit or broken wire. (2) An incorrect step, process, or data definition in a computer program.

Aside from minimal wording differences, it seems that the concept which the IEC vocabulary calls “fault” is called “failure” by the IEEE glossary, while the remaining term of the couple is treated rather differently by the two standards. Adding to the confusion, engineering parlance is demonstrably divergent from the standards. In a note, the IEEE glossary itself acknowledges that the definition of fault “is used primarily by the fault tolerance discipline” but that “in common usage, the terms ‘error’ and ‘bug’ are used to express this meaning”. Also the IEC admits that its definitions are somewhat at odds with common usage. In the “Terms and definitions” section of the recent standard on *Analysis techniques for system reliability* (IEC 60812, 2006), the definitions of failure and fault are taken literally from the IEC vocabulary but a note is added claiming that “for historical reasons” the two terms will be used interchangeably.

While, as far as the notion of failure is concerned, it is reasonable to see the differences between IEC and IEEE as mostly terminological, some authors have advocated for conceptual revisions. In discussing the notion of ‘software failure’, Chillarege (1996) argues that a more useful definition would read as follows:

The customer’s expectation has not been met and/or the customer is unable to do useful work with the product.

In the ensuing discussion, Yellman (1999) acknowledges that customer expectations should be preeminent, nonetheless he notices an important limitation in Chillarege’s definition: confronted with a product that demonstrably performs below specifications, engineers are willing to call it a failure, “whether or not they [the customers] think (rightly or wrongly) their expectations have been met”.

Although a few authors, like Chillarege, have proposed quite substantial reformulations, many authors have been more conservative and their proposals amount to a tailoring of the IEC definition to the needs of a specific domain. Being involved in machinery reliability and plant operations, Affonso (2006) claims:

Failure occurs when the component or equipment no longer can perform its intended function *safely*” (emphasis added).

Bauer et al. (2006), who write from the perspective of quality improvement, choose to define failure as follows:

Failure: the inability of an item, product or service to perform required functions *on demand due to one or more defects* (emphasis added).

Also interesting is the variant offered by Birolini (2007), which reads as follows:

A failure occurs when the item *stops performing* its required function” (emphasis added).

This characterization is noteworthy for, instead of focusing on the item’s “ability to perform” as in the IEC and IEEE definitions, it is centered on the actual observable performance of the item.

The engineering literature on failure seems subject to two conflicting demands. On the one hand, there is a quest for standardization and simplification; on the other, there is the acknowledgment of the multifaceted nature of failure phenomena which appears to resist a unique characterization. As a result, the literature is characterized by a dualism between a central core occupied by the most authoritative and well-established definition and a surrounding area composed of more specialized definitions. The center of the stage is occupied by the IEC definition, which is routinely considered “the traditional definition of failure” (Blache and Shrivastava 1994). Although this definition can deal successfully with a wide range of failure events, the proliferation of amendments and variations shows that there are circumstances which engineers are willing to describe as failures but are not easily captured by the traditional definition.

In the next section it will be shown that the traditional approach is based on four assumptions. Then, in “Beyond the Traditional Approach”, it will be argued that, in certain circumstances relevant for engineering practice, these basic assumptions may not hold.

## Four Basic Assumptions of the Traditional Approach

The traditional approach to failure can be analyzed in terms of four basic assumptions: *missing functionality*, *utilization context*, *item level*, and *negativity assumptions*. An event is an instance of failure in this approach when it satisfies all four assumptions.

The *missing functionality assumption* highlights the role played by the notion of an item’s function according to the traditional approach. The point is that items may behave in a number of aberrant ways and can possibly deviate from the specifications under several respects, but a failure is said to occur only when the performance of a *required function* is terminated, that is to say, it trespasses the acceptable limits.

Given the variety of function concepts available in the engineering literature (Erden et al. 2008; Houkes and Vermaas 2010), this assumption can be subject to multiple interpretations. Unfortunately, the IEC vocabulary does not offer much help in dispelling the ambiguity because the definition of required function it provides is circular. First, the vocabulary defines “required function” by means of the concept of “service” as follows:

Required function: a function or a combination of functions of an item which is considered necessary to provide a given service.

Then, the concept of “service” is defined by means of the term “function”:

Service: a set of functions offered to a user by an organization.

And, to close the circle, the definition of “function” is missing.

The paper by Rausand and Øien (1996) can shed some light on the meaning of function within the traditional approach. Instead of relying on the IEC vocabulary, they endorse the approach proposed by many design methodologists of articulating item functions by means of verb-noun combinations (e.g., “transmit signal”), which express the relationship between inputs and outputs of flows of energy, materials, and signals (Pahl et al. 2007; Stone and Wood 2000). Then, functions can be classified in various categories like *essential*, *auxiliary*, *protective*, and so on. Although Rausand and Øien do not mention explicitly the category of *required function*, they maintain that essential functions are precisely those “required to fulfill the intended purpose of the item” and are defined as “the reasons for installing the item”. Moreover, essential functions are reflected by the common names of the items themselves; for instance the essential or required function of a pump is “to pump a fluid”. Finally, they require that functions of items can be split into sub-functions and organized into functional hierarchies represented by so-called “functional block diagrams” as described, among others, in Pahl et al. (2007) and recommended by various technical standards as IEC 60812 (IEC 2006) and MIL-STD-1629A (1980).

In order to determine whether a failure has occurred, performance parameters have to be defined for all functions as well as target levels and acceptable limits. For an item like a pump, typical performance variables are pressure and flow rate. Hence, according to the traditional approach, a pump whose output pressure gradually dropped below the acceptable limit is said to have suffered a “gradual major failure”. In contrast, the gradual degradation of a sub-function that does not impair the essential function is called a “gradual minor failure”.

Second is the *utilization context assumption*. Utilization is the stage in the life of a product in which the item actually delivers its required function, and in doing so it fulfills the user needs. Typically, utilization begins once the product is installed and put into operation. A pump, for instance, is installed in a chemical plant with the purpose of pumping a fluid and then, for one reason or another, it stops doing so. This assumption is made more conspicuous and easier to spot in the version of the IEC definition given by Frawley (2002):

Failure: An event in which a *previously acceptable* product does not perform one or more of its required functions within the specified limits under specified conditions” (emphasis added).

Frawley’s definition implies that previously, at the beginning of its useful life, the product was performing at an acceptable level, after that its performance dropped and trespassed the acceptable limits.

Although the utilization stage is, of course, the natural context in which items are expected to perform their required functions, utilization episodes may occur in another context as well, namely during testing. Tests can be considered a vicarious form of utilization of the system they represent. Certifications tests, like those conducted on aircraft engines and mainframes, are extremely severe and aim “to test a complete technological system under conditions as close as possible to actual field conditions” (Sims 1999). Smaller scale and less demanding tests are routinely run after completion of the manufacturing processes or after maintenance operations. Even though the users, whose needs are supposed to be fulfilled by the performance of the item’s functions, are not actually present, their role is played by instruments and qualified personnel acting as vicarious users during testing. Therefore, as far as failure is concerned, tests can be seen as parts of a broadly construed *utilization context* which groups together utilization stage and tests.

Third is the *item level assumption*. When looking at the traditional approach as represented by the entire set of definitions in the IEC vocabulary, it can be seen that it rests on the idea of engineering products as physical items whose behavior can be observed and measured quantitatively. As shown above, failure judgments are based on the results of a comparison between the observed physical variables of a specific item (e.g., fluid pressure or flow rate of a specific pump) and the target values and acceptable levels defined by the specifications. In fact, as users we are surrounded and are dependent on the ability of many physical items to satisfy our needs by actually performing their required functions: cars move us around, clocks tell the time, printers print paper, and so on. Many of our judgments refer to the properties and behaviors of these items with which we deal on a daily basis.

Besides these judgments based on the properties of products at the level of the physical item, it is common for engineers, as well as for lay people, to make judgments also on properties at the type level. Reliability, for instance, is a property that can be predicated both of a specific item and of a type of product. The point is that the two kinds of judgments are different and the evidence that can be used in support of a judgment at the item level may be inadequate or insufficient for a type level judgment. So, at the type level a certain product, say the Volkswagen Golf, can be judged to be more reliable than a different but comparable type, say the Ford Focus; yet it is possible to claim, without contradiction, that a specific Ford Focus item has been found to be more reliable than a specific Volkswagen Golf.

In chemical engineering, criteria have been devised for assessing the safety characteristics of processing plants at the type level. For this kind of judgments, chemical engineers refer to the notion of inherently safer design (American Institute of Chemical Engineers 2009; Kletz 1998). Again, the judgment that a design is inherently safer is meant to describe a property, or a group of properties, that are not fully expressed at the level of the individual item. Instead, it is a judgment about features that are shared among all items realized following the same architecture.

Unless the notions of required function and observable performance are stretched beyond the characterization given by Rausand and Øien, the traditional approach is particularly suited for failure judgments at the item level and a broader notion of failure might be need for judgments at the type level (see “Beyond the Traditional Approach”).

Finally, there is a *negativity assumption*, that is to say, failure events are unwanted and should be avoided. The point of this assumption is that failures should be avoided *per se*, irrespectively of their consequences. Luckily only a minority of failures result in serious consequences and the vast majority cause only minor annoyances. Nonetheless, as remarked by Yellman’s (1999) criticism of Chillarege (1996) definition, engineers are willing to consider an underperforming product a failure whether or not the customers complain about it.

## Beyond the Traditional Approach

The view on failure embodied by the IEC definition and by the other definitions based on it has achieved a prominent status because of its undeniable agreement with a large class of events that are important in engineering practice. A great deal of engineering activity—during design, testing, manufacturing, maintenance, and so on—aims at preventing products in the field from terminating the performance of required functions. However, as it is shown by the existence of alternative definitions and by other evidence in the literature, engineers are prone to consider as failures also events that do not square with this interpretation; that is to say, events that do not satisfy the four basic assumptions.

The only assumption that most engineers will not consider challenging is *negativity*.^1^ They may disagree on whether an event or a class of events constitute a failure or not, but they will maintain that, if something is a failure, then it should have been avoided. In the rest of this section it will be shown how the first three assumptions may not hold in some circumstances that are relevant in engineering practice.

### Missing Functionality Assumption

In “Four Basic Assumptions of the Traditional Approach”, it has been shown that, according to the traditional approach, the notion of failure is binary: given a specific item and a specific (minor or major) function, either the item is functioning or it is not, depending on whether the observed performance lies within or without the acceptable limits. For simple items which are required to perform just one function there is nothing in between functioning as required and complete failure. Once the observed performance trespasses the acceptable limits, the items are said to be in a state of complete failure. In contrast, items performing multiple required functions can suffer partial failures, which can be minor or major depending on the importance of the affected function. Anyway, for a given required function (minor or major), the difference between functioning state and fault state is straightforward and it is based only on the observed performance being within or without the acceptable limits.

However, engineers appear to make use also of a more nuanced notion of failure, namely a notion that takes into account the rate at which the observed performance is approaching the acceptable limits and the residual ability of the item to perform. Lewis et al. (2003, p. 27), for instance, propose the following definition:

Failure is the inability of a product to meet or continue to maintain the performance or strength criteria in the application for which it was designed.

Therefore, an item whose observed performance is still within the acceptable limits could be classified as being in a fault state and removed from service because its performance is degrading at a rate much faster than predicted; that is to say, it is unable “to continue to maintain” the desired performance. If the same situation were to be assessed in accordance to the traditional approach, the verdict would be the opposite, for the judgment would be based on the fact that the item is still operating within the specifications.

An example of these different outcomes can be found in a case story discussed by Gagg and Lewis (2007) dealing with the failure of a swing bridge. The bridge moved over steel tracks laid in concrete, with the whole bridge riding on three castors, each one made of a steel shaft rotating within two bearing bushes manufactured from Oilon, a self-lubricating polymeric material. The bridge was in operation for 6 months and apparently was working as expected. Indeed, it was only during the first *routine* inspection that evidence of wear was found on the shafts of all three castors.

Despite that fact that, until the inspection, the bridge was performing as expected, Gagg and Lewis describe the three castors as the “failed axle bearing combination” (p. 1633). In fact, the castors were treated as failed components by removing them from service and by undertaking a detailed failure investigation. Eventually, it was determined that Oilon was not a suitable material for manufacturing the bearings and that the solution “to this ‘failure’ was a straightforward substitution of Oilon by Nylube, a grade of material far more capable of withstanding service loading experienced by this swing bridge castor” (p. 1634).

Items affected by accelerated wear are the typical subjects of failure judgments made in violation of the missing functionality assumption. Another common mechanism is corrosion, like in Suess (1992). Although several examples can be found in the engineering literature, they are less frequent than traditional failure judgments based on clear-cut termination of a required function because the latter are more conspicuous and potentially more harmful.

### Utilization Context Assumption

Although failures during utilization are usually the most worrying for engineers, they are aware that failure is lurking even before the products enter utilization and start delivering their required functions. Sudhakar and Paredes (2005) have investigated a case of “premature failure during manufacturing” (p. 35) affecting bimetal bearings for automotive application. In a manufacturing plant, a number of bearings were found to be cracked after the sintering step during which a layer of copper alloy was soldered to the steel backing. The analysis concluded that the bearing failures were due to improper setting of the heating parameters for the sintering process. It is clear that the traditional “termination of a required function” was not the criterion adopted by Sudhakar and Paredes when they claimed that the bearings suffered a “premature failure during manufacturing” and set out to identify the “failure mechanisms”.

### Item Level Assumption

According to the traditional approach, the notion of failure pivots on the idea of engineering products seen as physical items and on their properties and behaviors that can be observed and measured. However, engineers are used also to think in terms of properties of types of products, properties that do not exist at the level of the individual item. Consider, for instance, the notion of *field return* as it is used in the automotive industry. A field return is a car component that is returned to the manufacturer after a service technician has diagnosed a failure which is covered by the manufacturer warranty. In this sense, a field return is a physical item that has failed to perform its required function. Engineers, however, generalize this property over the entire type of a product, in which case it becomes the *rate of field returns* and, as such, it does not belong to any item in particular but to the entire type.

As its counterpart at the item level, also the type level property can be used in engineering judgments over failure. In the early 1980s, Ford introduced a new type of ignition module, the so called Thick Film Ignition module, with the purpose of surpassing the reliability of Japanese cars which were invading the US market. This goal was translated into a requirement to the effect that the rate of field returns must not exceed 1.6 returns out of 100 modules installed (Pecht 2006; Qi et al. 2008). After a few months, the ignition modules turned out to be a substantial failure with field returns so far above the expectations that Ford decided to issue a recall. The individual failures were due to a variety of reasons, including manufacturing defects, assembly problems, and inappropriate maintenance. However, the main reason for the spate of field returns was the underestimation of the temperatures prevailing in the operational environment, that is to say, the engine compartment where the modules were installed. Unable to withstand the thermal loads, the modules behaved erratically resulting in what “may well be the most widespread intermittent failure condition ever reported” (Qi et al. 2008, p. 664). Sure enough, many individual modules did operate successfully: those were the modules installed on cars operated in colder regions. The point is, however, that, as a type, the Thick Film Ignition module was unable to achieve the expected rate of field returns.

The Ford case deals with a type level property of a component, the ignition module, which is part of a larger system, the car. Engineers, however, express judgments also on the basis of type level properties of entire systems. Marks (1989) offers an analysis of the Sinclair C5, a battery-assisted tricycle which was conceived by the British inventor and entrepreneur Sir Clive Sinclair and was introduced to the market in January 1985. The sales figures were so poor that production was stopped as early as September 1985. After reviewing the main steps in the development, launch, and failure of the C5, Marks is led to wonder: “but was the C5, in fact, a marketing failure or a technology failure?” In Marks’ opinion, both the product and the marketing were poor. As for the “technology”, Marks notices that the product can be criticized for a series of shortcomings relating to aspects like safety (e.g., low protection in case of collisions) and usability (e.g., lack of a reverse gear). It is clear, then, that Marks’ judgment does not refer to any C5 in particular for failing to perform its required functions; instead the criticisms point to crucial properties of the C5 as a type. The manufacturing quality and reliability of the physical items might have been excellent and many customers might have enjoyed riding it; yet, as a type, it was an uncontested failure.

The traditional view is not well equipped for this kind of failure judgments that are based on type level properties emerging from the interaction of multiple aspects of a product design and life cycle. Such properties like inherent safety or robustness or sustainability, especially in case of complex products, cannot be easily reduced to a specific function or small group of functions expressed as input and outputs in the functional hierarchy of a product. Still, type level characteristics are crucial for the realization of successful products and play an important role in the delineation of product requirements.

Admittedly, the distinction between functional and non-functional requirements is hotly debated in the requirements engineering community (Hull et al. 2010). In fact, the definition of requirement given in the IEEE 1220 (2005) standard carefully avoids introducing the notion of a non-functional requirement claiming, instead, that a requirement is “a statement that identifies a product or process operational, functional, or design characteristic or constraint”. In turn, the notion of a design characteristic covers a wide spectrum of “performance, usability, safety, maintainability and a host of other qualities” (Hull et al. 2010, p. 7).

It is worth stressing that the argument developed in this paper in support of a broader notion of failure does not depend on the availability of a shared and clear-cut distinction between functional and non-functional requirements. What is needed is the acknowledgement that the traditional notion of failure is based, among others, on the assumption that functions of engineering products should be expressed by means of input–output relations of flows of energy, materials, and signals that can be organized in functional block diagrams. Many design methodologists, e.g., (Pahl et al. 2007; Stone and Wood 2000), have convincingly discussed the various benefits allowed by this interpretation in support of the engineering design task, as well as in comparing, communicating and archiving design solutions. Nevertheless, nowhere is it implied that, in order to achieve these benefits, this interpretation has to cover all relevant aspects of engineering products. On the contrary, it can be expected that an overly generic notion of function would undermine the benefits that have just been mentioned.

Thus, the main purpose of this section has been to show that products can have characteristics which engineers themselves deem relevant for failure judgments and that cannot be captured easily in terms of functions and functional hierarchies. Since engineers routinely make these kinds of judgments and the traditional approach appears to struggle with them, a more efficient way of dealing with this conceptual conflict is to devise a broader notion of failure. The new notion of failure will be introduced in “A New Definition of Failure”, which will also explore how the new notion connects to the life cycle perspective of product development. Before that, the next section will further investigate the conceptual background of the traditional approach to failure and its connection with the sequential model of product development.

## From One Customer to Many Stakeholders

The fact that there are problematic cases does not imply that the traditional approach is unwarranted, for it does provide an adequate representation of many kinds of failure events that engineers recognize as threats to the success of their products. Moreover, it sits well with widespread intuitions of end-users. The question then, is why the traditional approach has left out the problematic cases from the mainstream of failure events.

The analysis performed in the previous sections suggests that this exclusion depends on the fact that the traditional approach is based on a specific interpretation of the mission of engineering product development. Roughly stated, this mission can be described as follows: to develop products that provide optimal and reliable performance of required functions for the customer. Then, the traditional approach assumes that, as far as the “required functions” are concerned, the customer and the end-user are considered to be the same; that is to say, the individual or organization whose needs are satisfied by the functional performance of the product.

It has to be stressed, though, that the identification of end-user and customer may not hold for product characteristics that are not considered strictly functional. This point can be illustrated by the following historical example. In the early 1920s, during the first stages of the development of household refrigerators, General Electric engineers had to decide how to solve the problem of cooling the compressor, that is to say, the component that circulates the refrigerating fluid (Cowan 1985). They came up with two alternatives: water cooling or air cooling. Both solutions were thought to be equally capable of contributing to the achievement of the required function of a refrigerator, that is, to keep food items fresh. The crucial difference between the two solutions was energy consumption. According to the calculations “the electric power bill of the air cooled machine would be about $ 1.30 more in 6 months than the water cooled machine” (p. 209). Since electric utility companies were General Electric’s most important customers, the air cooling mechanism was selected. Of course, in those days there were no standards or energy saving regulations to deter General Electric from its decision. Nevertheless, it is reasonable to assume that also within more tightly regulated markets, all else being equal a company will prioritize those non-functional characteristics that are more beneficial to its customers.

Although this interpretation of the mission statement is still extensively shared among engineers and managers alike, other interpretations (that will be discussed later in this section) have been proposed that take into account the needs (both functional and non-functional) of multiple stakeholders involved in the life cycle of a product, thus prompting more liberal views on failure.

### The Sequential Model of Product Development

It is worth stressing that the emphasis on the end-user is far from being idiosyncratic and disconnected from practice; on the contrary, it can be considered part of the conceptual background of the so-called “sequential model” of product development (Kahn 2005; Yang 2007).

In this model, the overall design task is broken down into sub-tasks that are carried out by individual departments in a predefined sequence. The details may vary, but generally speaking the process starts out with the identification of a series of needs that the product is expected to address; the needs are translated into requirements, and from the requirements a design concept emerges that is progressively made more precise until it is approved for production. The upstream decisions taken early on in the process have the effect of freezing the main features of the final architecture and, at the same time, of dictating the boundaries or the framework within which downstream departments will have to carry out their tasks and advance the process towards completion.

The framework plays also the role of a guideline for the assessment of what kinds of product behavior count as a success or as a failure. Therefore, since the framework relies on the list of functional requirements resulting from the analysis of the user needs, it prompts for the endorsement of a notion of failure that assumes the performance of required functions as the focal criterion.

A persistent worry in the analysis of user needs is the anticipation of potential behaviors that might result in product misuse. Although, practically, products cannot be designed to survive all conceivable kinds of abuse, to a certain extent engineers can control and reduce the probability of misuse causing a product to “fail” in a dangerous manner. Here the term “fail” is between quotation marks because, as noted by Ezrin (1996), products that break apart “when someone makes […] improper use of them, that should not be considered failure in the usual sense”. Still, engineers are typically aware of the duty to minimize the probability of harm also for “failures” due to misuse, and may take it into account during the design process in the form of implicit user needs.

The sequential model has been the paradigm for product design until the 1970s, when industry started switching towards alternative models based on the promotion of cross-disciplinary integration which proved to be instrumental in the commercial success of Japanese manufacturers, particularly in the automotive sector. Nowadays, the sequential model can still be effective for the development of mature products where companies have already extensively explored many kinds of different frameworks in a number of product generations (Liker et al. 1996). Hence, downstream departments are equipped with a large repertoire of solutions that can fit with almost every possible framework.

One of the main shortcomings of the sequential model is that costs of major changes can increase exponentially as the product development proceeds to the later phases. Major changes are those demanding a revision of the framework. If, towards the end of the process, e.g., close to the manufacturing stage, a sub-task cannot be completed and the design is sent back to the drawing board, then all the downstream decisions have to be checked again to assure they are compatible with the revised framework. Even in the ideal case that most of the downstream decisions can be safely retained, it is likely that delays will ensue. Taking problems related to reliability as an illustration of the extent of these risks, Levin and Kalal (2003) have estimated that “the cost to fix a reliability problem increases an order of magnitude in each subsequent phase”.

Research on design methods has also pointed out that alternative models, besides curbing the cost of late stage design revisions, may allow for a host of other improvements, like more robust designs, shorter development times, and exploration of a broader range of design solutions (Henderson and Clark 1990; Clark 1991; Iansiti 1995; Hoopes and Postrel 1999).

For these reasons, ever more engineering companies are switching towards integrated approaches to product development inspired by the example of Japanese car manufacturers like Toyota (Womack et al. 2007). Evidence for this transition has been provided by several studies (McDonough 2000). Griffin (1997) presents the results of a 5 years research effort showing how new product development relies increasingly on multi-disciplinary or trans-disciplinary teams. Helper and Sako (1995) focus on the change in the customer–supplier relations and find that “where once contracts were short-term, arm’s-length relationships, now contracts have increasingly become long term” (p. 77) providing better sharing of information and cooperation.

### Integrated Models

Several alternative models have been offered, like Concurrent Engineering (Syan and Menon 1994), Simultaneous Engineering (Kortge and Okonkwo 1989), Integrated Product Development (Andreasen and Hein 1987), and the literature on the subject is flourishing. One aspect these models have in common is to look at the product and the processes taking place during the product’s life cycle as an integrated system and to emphasize the interdependencies both across components and across processes. Since, in one form or another, the notion of product life cycle plays a crucial role, the various integrated models on offer can be seen as interpretations of a general life cycle perspective to product realization. In the words of the recent ISO/IEC standard *Systems and Software Engineering* (ISO/IEC 15288, 2008), the aim of a life cycle perspective is to provide a “common framework to improve communication and cooperation among the parties that create, utilize and manage modern systems in order that they can work in an integrated, coherent fashion”.

At the foundations of the life cycle perspective is the observation that every system has a life cycle which can be represented by means of a *life cycle model* constituted by a sequence of stages, from concept development to retirement. The number and kind of stages employed vary depending on the nature of the product to be realized and the structure of the organization. A life cycle model is not a mere chronological representation of the typical development of single items of the product, although such representation can be easily derived from the model. Instead, it is a “decision-linked conceptual segmentation” (ISO/IEC TR 24748-1, 2010) used to represent and manage technical and business decisions and actions during the life of a product as a whole. The stages in a life cycle model perform two main functions: they group together homogeneous technical and managerial activities; and also provide a systematic view of the requirements that the product must be able to achieve in order to be approved for the following stages.

The key difference between the sequential model and the integrated models can be appreciated already from the early stages in the product development process. In the former, designers are in charge of translating the end-user needs into requirements and, in doing so, establishing the framework for the activities downstream. In the latter, a trans-disciplinary team is invested with the responsibility of integrating the view of the various subjects having stakes in the different stages of the life cycle. Hence, in a trans-disciplinary team, the aim of the designers to define the functions that the product is requested to perform during utilization meets with the aim of the manufacturing engineers to optimize the processes on the shop floor, and with the needs of component suppliers, and with the demands of the maintenance and support department, and so on.

Instead of being dominated by a list of requirements connected to the functions (allegedly) required by the end-user, an integrated product development process is led by a trans-disciplinary set of goals whose aim is to strike the balance between the many stakeholders involved in the whole life cycle. As product development unfolds the set of goals is increasingly refined and made more precise by exploring and expanding it into requirements and finally into technical specifications. However, in order to assure that the goals are preserved, this process is always supervised by the trans-disciplinary team.

From a life cycle perspective, the mission of engineering product development is broader than the one envisioned by the sequential model because there is not just one customer, the end-user, to whom an optimally and reliably performing product should be provided, but there are multiple stakeholders having different—and sometimes conflicting—interests in one or more of the stages in the life of a product. Both the notion of successful product and that of failed product are affected by the life cycle approach. In fact, to be fully successful a product must satisfy end-user needs by performing its required functions during utilization. However, a product that performs well in the field but falls short of meeting the demands of other stakeholders, e.g., the manufacturing department, may result in scarce profitability and, eventually, in failure. What characterizes the life cycle approach to failure is precisely the awareness that a life cycle is an integrated whole in which the needs of many stakeholders have to be balanced in order to avoid failure.

Now, if the traditional definition of failure sits well with the sequential model, then a suitable definition should be identified that suits the needs of the life cycle approach. In the next section, a few alternative definitions will be examined and a new definition of failure will be introduced and it will be shown that, besides dealing successfully with the failure events typically addressed by the traditional approach, the new definition can also accommodate the problematic cases.

## A New Definition of Failure

A number of attempts have been made to extend the reach of the traditional definition, but they have been only partially successful. One avenue is to link the notion of failure to the product specifications as done, for instance, by the US Military Standard (MIL-STD-721C 1981) *Definition of Terms for Reliability and Maintainability* where the following definition is given:

Failure: the event, or inoperable state, in which any item or part of an item does not, or would not, perform as previously specified.

The problem with such kind of definitions is that product specifications may turn out to be inadequate thus leading to products that fail although they do comply with the specs. Groot Boerle (2002) describes the case of an electric wheelchair that went out of control, drove off a subway platform and badly injured the driver. The investigation established that the wheelchair was activated by a low-voltage electromagnetic field at a frequency of 1.89 GHz, which is one of the frequencies used by digital telephone networks. During the ensuing trial, the manufacturer declined responsibility claiming that the chair met the product specifications, according to which the electrical system had to be insulated from electromagnetic fields up to 1 GHz. This line of reasoning was rejected by the court on the grounds that the specifications for a wheelchair supposedly fit for utilization in public spaces should take into account the possibility of interferences with telephone networks.

A second alternative for broadening the reach of the traditional approach consists in emphasizing the role of product requirements instead of that of product specifications. Also this fix, however, runs into the same kind of problems because it falls short of capturing situations in which product requirements themselves are misguided, as exemplified by the Thick Film Ignition module developed by Ford (see “Item Level Assumption”). The requirement asked for modules able to withstand continuous exposure to temperatures up to 125°C (Pecht 2006), which was assumed to be the hottest temperature in the area of the engine compartment where the modules were to be installed. The requirement turned out to be unrealistically low, leading to the spate of field returns that prompted the recall.

Since the amendments discussed above and other proposals available in the engineering literature have been unable to reshape the traditional definition such that it can deal with the life cycle approach, a new definition of failure is offered in this paper.

The purpose of the new definition is to capture how engineers currently use the notion of failure. The previous sections have shown that this use is broader than the range covered by the traditional approach. Moreover, the expanding range of the notion of failure appears to be correlated with the larger set of issues that are dealt with in life cycle approaches to product realization. Therefore, the new definition has been conceived by having in mind the kind of failure judgments and concerns about failure that may emerge within trans-disciplinary teams involved in product development. At the same time, the new definition has to be backward compatible and retain the ability to address the failure judgments that are the domain of the traditional approach. In order to underline the difference from the traditional definition, the new notion has been baptized *product failure* and it is defined as follows:

Product failure is the inability of an engineering process, product, service or system to meet the design team’s goals for which it has been developed.

Visibly, the structure of the new definition is remarkably similar to the traditional definition and retains the basic intuition that failure is a form of inability on the part of the product. The novelty is due to the fact that, instead of referring to the notion of required function, the intuition is anchored to the frame provided by the life cycle approach to product development by creating a connection with “goals of the design team”.

While the idea of product life cycle has been a main source of inspiration for the new definition, the notion of life cycle does not appear explicitly in the text of the definition. The reason is that, although a life cycle approach to product development and, consequently, the adoption of trans-disciplinary teams is becoming increasingly popular, it is unlikely to become the exclusive approach to product design. As noted above (“The Sequential Model of Product Development”), the sequential model is still effective in design of mature products. Also, not all design groups are necessarily trans-disciplinary. Therefore, to make the notion of product failure as general as possible it has been decided to phrase it in terms of “design teams”. When it is applied within the context of the sequential model, the new definition converges with the traditional approach where the goal of design teams is to develop products that satisfy end-user needs by performing required functions. When it is applied from a life cycle perspective, design teams become trans-disciplinary and their goals result from the integration of needs and concerns of multiple stakeholders.

It is worth noticing that trans-disciplinary teams may gather together members coming from different companies, like suppliers and contractors, in addition to representatives of the company owning the product. Hence, the set of goals on which the design team will settle can be seen also as an integration of the goals of the various companies or organizations taking part in the effort. So, the locution “design team’s goals” is more general than “company’s goals”.

Yet, even though a life cycle approach to product realization broadens the kinds of concerns that are taken into account during the design process, the design team’s goals cannot be expected to converge with the needs and priorities of society at large. Even in a life cycle approach, product development and realization remains a technical activity pursued predominantly for economical purposes. Certainly, economic viability presupposes compliance with the law and the relevant regulations, which are the most prominent expression of the needs and priorities of a society. But when engineers look at a product to assess whether it is successful or not, they think it is possible to tell apart its technical merits and other non-technical properties. The adoption of a life cycle perspective has expanded the domain of the technical merits beyond the mere ability to perform required functions, but has not changed the fact that product development is a technical endeavor for the benefit of a well-defined set of stakeholders. Indeed, a conflict of intuitions may ensue, as it is illustrated in the following case history. As mentioned above, the development of new cars in the US automotive industry follows the integrated model and relies heavily on trans-disciplinary design teams. Nonetheless, one of the main recent innovations in the US market was the introduction of Sport Utility Vehicles (SUV) which, although extremely profitable for the industry, has raised many serious social and moral issues. Large and luxurious SUV generate more profits than any other type of car. In the early 1990s, Ford was making less than $1,000 in profits on the average sedan, while the profit on large SUV models like the Explorer was nearly $8,000 (Bradsher 2000). On the other hand, SUVs are demonstrably more polluting and more dangerous than average cars; especially in multivehicle collisions, their mass and the stiffness of their framework cause a substantial increase of the likelihood of severe injuries and casualties (Bradsher 2002; Latin and Kasolas 2002). These social costs, however, never played a prominent role within the definition of the design goals: as recently as 1997, Ford’s Director of Vehicle Systems Engineering conceded that “crash compatibility was not an active part of design” (Bradsher 1997). According to the definition given in this paper, SUVs like the Ford Explorer cannot be considered a “product failure”, even though there might be compelling reasons to consider them examples of dangerous kinds of products.

It cannot be overstressed that satisfaction of the end-user needs through performance of the required functions is a crucial element of the set of goals of any design team. Thus, the new definition is in agreement with the traditional one in that termination of the ability to perform a required function constitutes a failure event. Moreover, it inherits the view that for failures to occur it is not sufficient that performance variables deviate from target values, but acceptability limits should be trespassed. Nonetheless, the new definition has a broader reach. Being centered on the *goals* of the design team, the concept of product failure can deal with the cases of wrong specifications and wrong requirements discussed above. Considering again the example of the electric wheel chair, it can be seen that it was indeed a case of product failure because the main goal behind the realization of an electric wheelchair is to make a product that can be safely used for transportation on public roads. Vulnerability to interferences from telecommunication networks makes the product unsuitable for the intended application. Similarly, products may be produced which are in compliance with the requirements, but the requirements themselves may be an inadequate expression of the design goals (e.g., Ford’s ignition modules). These situations are also covered by the notion of product failure.

The most important aspect of the new definition is how it deals with the traditional approach and the stance it takes towards its assumptions, i.e., missing functionality, item level, utilization context, and negativity assumptions. It can be shown that, besides the negativity assumption, on which they are in agreement, the two approaches take different stances.

First of all, the notion of product failure does not maintain the missing functionality assumption; therefore a product that is still performing its required function but is falling short to achieve the goal for which it was developed, is classified as a failure. Thus, the notion of product failure can capture cases of failure like the swing bridge investigated by Gagg and Lewis (2007) (“Missing Functionality Assumption”). Even though the bridge was still performing its required function, it was unable to meet the goals of robustness and prolonged operational life.

Secondly, the notion of product failure can accommodate cases of failure that do not comply with the utilization context assumption (“Utilization context assumption”). The bimetal bearings that developed cracks during manufacturing can be considered an instance of product failure because one of the design goals is to assure that products can be successfully and reliably manufactured.

Finally, since a trans-disciplinary development team may set goals for the product as a type and is not confined to item level goals, the notion of product failure does not rely on the item level assumption. Therefore, the inability of the Ford ignition modules to achieve the expected reliability goals (expressed in terms of field return rate), which proved to be problematic for the traditional approach (“Item Level Assumption”), is correctly classified as a type level failure.

Before the analysis moves to further aspects of the life cycle approach, it may be worth summing up the main steps in the argument developed so far. It has been argued that, although the concept of failure is variously defined in engineering, a traditional approach can be identified which can deal with a large proportion of failure events that matter to engineers. However, a number of problematic cases have been shown in which the assumptions of the traditional approach are at odds with engineering intuitions and ways of speech. The explanation of the inability to account for these violations has been identified in the relation between the traditional approach and the sequential model of product development, from which the traditional approach has inherited a bias in favor of the end-user perspective. This model has been contrasted with integrated models of product development that are based on a life cycle perspective and emphasize the role of a multiplicity of stakeholders besides the end-user. Then a new definition of product failure has been proposed based on this life cycle perspective: product failure. Finally, it has been shown that, besides addressing the failure events covered by the traditional approach, the new notion can deal also with the problematic cases.

In may be worth pausing briefly to consider whether, by looking for the advantages of a broader notion of failure, this paper has ended up propounding a definition that is overly stretched. More specifically, a concern may be raised that the definition of product failure is dangerously close to becoming a synonym of design flaw, that is to say, any reason for design iteration. Although there are similarities between the two notions it can be shown that they are still clearly distinguishable. First, product failures can be addressed without recurring to design alterations. When Apple announced the new iPhone 4 back in June 2010, it claimed that the phone would have been available in two colors, black and white. However, even though the black version arrived as anticipated, the white version was delayed without further explanation, and only at the end of July Apple confessed that the white phones turned out to be “more challenging to manufacture” than expected (Apple Inc., 2010). The company carefully avoided to specify the nature of the manufacturing challenges. Technology experts suggested that the delay was due to problems faced by the supplier of the white glass panels that constitute the front and back covers of the handset (Lai 2010). Although the supplier was able to manufacture the prototypes according to the tight opacity and thickness specifications, when ramping up to full scale production the manufacturing yield fell dramatically with merely three panels fabricated successfully per hour (My Digital Life 2010). The problem was not solved until, in January 2011, Apple found a new supplier able to achieve an acceptable yield while meeting the specifications. Eventually, the much awaited white models reached the shops at the end of April 2011. There was no design iteration, because the design as well as the product specifications were retained and the new supplier proved that they could be achieved. Nevertheless, the experts maintain the product was a failure because many prospective customers postponed their purchase and decided to buy the next version of iPhone that was launched in October 2011 (Sherr 2011).

Second, design iterations may occur that are not due to product failures. Changes in regulations (e.g., environmental protection laws) or in the financial situation may force a team to alter an otherwise sound design.

Moreover, the definition of product failure appears to be well suited for being adopted in the execution of Failure Modes and Effect Analysis (FMEA), a design procedure for the systematic identification of potential failures that is widely used in industry and recommended by international quality standards and best practices such as (ISO 9001, 2008) and (SAE J1739, 2002). In his well-known manual on FMEA, Stamatis (1995) emphasizes that the purpose of the procedure is not merely to identify and prevent failures that might occur once the product enters the utilization stage; instead, it should also be implemented to address failures located elsewhere in the life cycle. For this reason, Stamatis distinguishes four types of FMEAs as follows: Systems FMEA is based on conceptual design and focuses on potential failure modes between the functions of the system; Design FMEA is used to analyze products before they are released to manufacturing and focuses on failure modes caused by design deficiencies; Process FMEA aims at minimizing process failures during manufacturing and assembly; finally, Service FMEA is used to analyze services before they reach the customer. Even though the four types of FMEA are based on different sources and address different issues, Stamatis proposes a unified definition of failure, which is the following:

Failure: the problem, concern, error, challenge. The inability of the system, design, process, service, or subsystem to perform based on the design intent. (Stamatis 1995, p. 74)

Since he is writing a manual for practitioners, it is not surprising that Stamatis decides to keep the conceptual analysis to the bare essentials by simply stating his definition without expanding into the motivations behind his choice. However, two crucial clues are given that highlight the similarity between his notion of failure and the one discussed in this paper. The first clue is found in the brief comment that follows the definition explaining that the design intent “usually comes from an analysis and an evaluation of the needs, wants, or expectations of the customer” (p. 74). The second clue is a remark from the beginning of the book stressing that the customer should not be interpreted just as the end-user, instead it should be “viewed as the subsequent or downstream operation as well as a service operation” (Stamatis 1995, p. xxiii). Jointly, these two clauses allow for Stamatis’ notion of failure to span over the same range of failure judgments that led to the notion of product failure analyzed in this paper.

## The Life Cycle Approach in Action

**Fig. 2:**
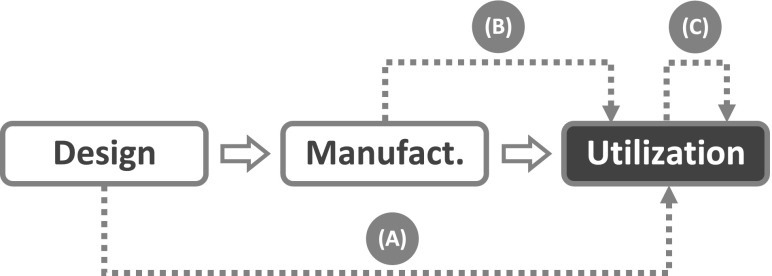
Admissible failure trajectories according to the traditional approach: *arrow* (*A*) design failure; *arrow* (*B*) manufacturing failure; *arrow* (*C*) utilization failure

Given the discussion above, it will come as no surprise that the term *life cycle* does not appear among the terms defined by the IEC vocabulary (IEC 50(191), 1990). What can be found, instead, is an implicit reference to the concept of life cycle in relation to a small group of definitions dealing with the causes of failure. *Failure cause* is defined as “the circumstances during design, manufacture or use which have led to a failure”. The analysis by IEC vocabulary stops here: there are no further considerations on the life cycle stages and the relations between them. And here is where the life cycle approach takes over. The first step consists in making explicit the life cycle model assumed by the IEC analysis, as it is done in Fig. 2. Then, the next step consists in representing the three circumstances “which have led to a failure” as *failure trajectories* which connect the stage where the causal factor lies with the stage where the failure event is located.

Since Fig. 2 has been built using the concepts and definitions available within the traditional approach, only three failure trajectories can be represented, all of them point to the utilization stage, and the life cycle model stops with the utilization stage. But these limitations do not apply to the life cycle approach. Since the needs of other stakeholders besides the end-user are taken into account (e.g., those of subjects having stakes in stages like installation, shipment, maintenance, recycling, and so on), more sophisticated life cycle models can be introduced which extend beyond the utilization stage (see below).

**Fig. 3:**
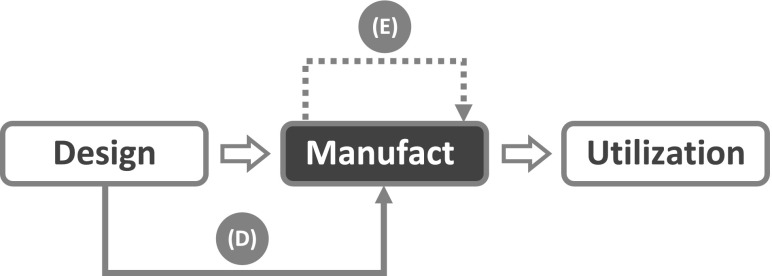
Failure trajectories of product failures located at the manufacturing stage

Moreover, the withdrawal of the utilization context assumption implies that more failure trajectories can occur that are not bound to point to the utilization stage. It can now be seen that the life cycle approach can easily accommodate the case of the bimetal bearing failing during manufacturing discussed in “Utilization context assumption”. Although the failure did not occur during the utilization stage nor involved a termination of a required function, according to the life cycle approach it constitutes an instance of product failure because those individual items that cracked during the sintering process were unable to meet the quality characteristics which were part of the design goal. Since the failures were due to a causal factor associated with the manufacturing stage itself (i.e., inadequate setting of sintering process parameters), the corresponding failure trajectory can be represented by an arrow like (E) in Fig. 3, which starts out from the manufacturing stage and then points to the same stage, where the failure is located.

Figure 3 displays also a second trajectory, arrow (D), which connects the design stage with the manufacturing stage. As the abundant literature on Design for Manufacturing and Design for Assembly shows, not all design solutions are equally easy or convenient to manufacture, and some may be unfeasible altogether. Although designs are checked for features that may hamper manufacturability, it can happen that a design is approved for production even though it contains a flaw resulting in the inability of the product to run through the entire process or, in case it can make it, in the inability to meet the acceptability requirements. Both outcomes are to be considered product failures.

Failures during manufacturing can be no less consequential than during utilization. This is especially the case in civil engineering. A notable historical case is the failure during construction of the Quebec Bridge (Quebec City, Canada) in 1907. The investigating commission established that several design shortcomings were responsible for the collapse, among which were the “use of relatively slender struts with an inefficient layout of material, calculations using methods derived from much smaller sections, inadequate lacing, [and] over-stress due to inaccurate dead loads” (Collings 2008). As a result, the bridge was structurally unable to withstand its own weight. The introduction of more powerful engineering models and computational tools has greatly reduced the occurrence of similar failures, though not completely. In June 1970 a section of the Cleddau Bridge (Neyland, UK) collapsed during construction and the Committee of Inquiry determined that inadequate design of a pier support was a crucial factor (Collings 2008; Merrison 1973).

Analogous mishaps, albeit with less dramatic consequences, may occur with any kind of engineering artifact. Consider the manufacturing of tempered glass: tempering is a thermal or chemical treatment that confers to glass an increased strength compared to normal glass and it is used in the realization of products like windows, doors, tables, and building facades. The improvement of mechanical properties is due to the rapid cooling which places the surfaces of the glass in a state of high compression, and the central core in a state of compensating tension (Pfaender 1996). As a result, tempered glass is about four times stronger than normal glass of the same thickness. Moreover, when it breaks, tempered glass fractures into small fragments of nearly regular shape that reduce the probability of serious injury as compared to normal glass.

The downside is that, if the internal stresses between glass surface and central core are not carefully balanced, they can build up during the tempering process and shatter the glass to pieces. Therefore, the designer must be aware of a series of geometrical constraints that are dictated by the need to balance internal stresses. If holes are needed in the final product (for instance, to allow the installation of metal hinges on a glass door) their minimal diameter should be not less than the glass thickness, and the distance between the holes should be at least four times the glass thickness (Le Bourhis 2008). Lack of compliance with these constraints will result in a product bound to fail during manufacturing because of inadequate design, arrow (D).

Figure 3 can also be used for presenting a third aspect that tells the life cycle approach apart from the traditional approach, which is the possibility of distinguishing from failure trajectories at the type level and at the item level. In Fig. 3, the dashed arrow (E) represents an item level failure trajectory; the solid arrow (D) represents a type level trajectory. At first, it may be tempting to differentiate item level failures and type level failures looking at the number of items involved: just one in the former, all the items in the product population in the latter. Although this may be correct in most circumstances, it is misleading. According to this criterion, the collapse of the Quebec Bridge was just an instance of an item level failure that happened to be also a type level failure because the entire product population was constituted exactly by that precise item. However, the results of the Commission of Inquiry made it clear that there were serious problems with the bridge design such that any further attempt to rebuild it would end in a new collapse unless adequate changes were made. Precisely this link with design solutions is the aspect that characterizes type level failures. They are due to design flaws and the proper way they can be corrected or prevented is by design improvements. Certainly, changes to the manufacturing process can sometimes rescue a bad design, but those are usually mere temporary fixes and, if not properly thought through, are likely to result in other unforeseen problems in later stages. Also relevant for type level failures is the fact that, routinely, designers set goals dealing with type level properties of products. An example is the reliability goal that Ford engineers set for the ignition modules (“Item Level Assumption”), namely that field returns should not be in excess of 1.6 out of 100 components threshold. Clearly, this is a reliability property that cannot apply at the level of the individual item, and deals with properties pertaining to the type as a whole. Another example of type level goal is manufacturing yield, which expresses the ratio of acceptable items within the overall manufacturing output.

In contrast, item level failures are due to process variables (e.g., manufacturing, utilization, maintenance variables) that typically affect only a limited number of items, and can be prevented by means of a correct adjustment of those variables. In the case of the bimetal bearings (“Utilization context assumption”), for instance, the modification of the sintering timing and temperatures was successful in eradicating the problem. Since item level and type level failures are remarkably different in terms of consequences and of corrective actions, it useful to distinguish them graphically when drawing the failure trajectories in a life cycle model. Since design flaws result in type level failures, the arrows starting out from the design stage are represented by solid arrows. The failure trajectories originating from the subsequent stages, which result in item level failures, are represented by dashed arrows.

The same line of reasoning that has been applied in the analysis of the potential failure trajectories represented in Fig. 3 can be extended to more detailed and complete life cycle models; that is to say, models that represent sub-stages within the main stages (e.g., milling and welding within manufacturing), or models that include stages which occur after utilization. In fact, the life cycle approach introduces the possibility of analyzing failures that can occur also after a product has completed its useful life, a possibility that is not contemplated by the traditional approach. In particular, the need of minimizing the environmental impact of products, a need that is backed up by increasingly stringent regulations, implies that the design intent is informed by the requirements of stakeholders whose interests lie in the environmental performance of products during the retirement stage.

**Fig. 4:**
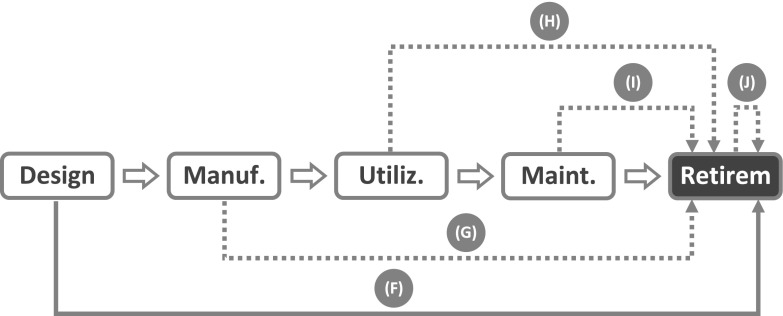
Product failure trajectories related to the retirement stage of an electronic appliance

What are, then, the potential failure trajectories that point to the retirement stage of a product? Let us consider an electronic appliance for which the designers aim at minimizing the environmental impact during retirement. The product life cycle can be represented, as in Fig. 4, by a model constituted of five stages: design, manufacture, utilization, maintenance, and retirement.

The goal of environmental optimization evolves into a series of requirements for the retirement stage that set the acceptable limits for a range of relevant parameters like, toxic emissions, recycling of materials, reutilization of components and so on. The ability to achieve these requirements depends on decisions and actions taken during each of the stages within the life cycle. A way in which design can increase the recyclability performance is by reducing the number of small plastic components within the appliance. In a study on design for recycling of computer enclosures, Masanet and Horvath (2007) have shown that “PC enclosure components with a mass of 25 g or less would be discarded (a common practice for small plastic components)”. The discarded components detract from the recycled fraction and can cause the product to miss the established goal, thus leading to a product failure during retirement due to design related factors. This failure trajectory appears as arrow (F) in Fig. 4. Differently from the other trajectories, this failure occurs at the type level; hence it is represented by a solid arrow.

Manufacturing variables can determine item-level product failures during retirement, arrow (G). Typically, recycling of the printed circuit boards installed within electronic appliances is done through converters in which the plastic part is burned and the metals are recovered. One potential environmental risk is posed by the presence of toxic additives that are sometimes utilized in the manufacturing of the plastic part. By careful selection of materials and control of the manufacturing processes, the amount of toxic additives can be minimized. It may happen, however, that because of a mistake during manufacturing a number of plastic parts are produced which contain a large amount of toxic additives that will be released during recycling, thus leading to a violation of the environmental requirements.

Arrow (H) represents product failures due to circumstances related to the utilization stage. A typical example is the failure to achieve predetermined recycling goals because users, instead of returning the products to the appropriate service centers, simply dispose of them. As documented by Behrendt et al. (1997) this is more probable for small appliances that are normally disposed of with household waste.

Maintenance procedures, arrow (I), can alter a product in such a way that it becomes unsuitable for disassembly, e.g., bolted joints are replaced with welded or glued joints. Finally, arrow (J), variables related to the retirement stage itself, like incorrect temperature settings of the converter, can result in failure to achieve the design goals.

Although extremely simplified, the example above suggest the ability of the life cycle approach to identify and methodically represent a wide range of circumstances that may cause a product to miss the established design goals. Sure, the idea of product failure is intuitively more easily associated with stages like utilization or manufacturing than with the retirement stage. However, restricting failure to certain stages would undermine the life cycle approach according to which failure can be found anywhere in the life cycle.

So far, only direct failure trajectories have been examined, that is to say, trajectories originating in one stage (e.g., design) and then pointing directly where the failure is located (e.g., utilization). Engineers, however, routinely have to confront with more complicated scenarios in which multiple factors may be involved and intermediate stages also play a role. As an example, let us consider a failure case analyzed by Barella et al. (2011). A large batch of canned tuna in olive oil (1 million cans) failed during the utilization stage, approximately 6–8 months after production, when customers started complaining because the product appeared to be contaminated. The sources of contamination were oxidation products developing near the welding area of the can. Typically, tuna cans are produced by welding sheets of tin coated steel along one edge. As an additional protective measure, after welding a polymer coating, i.e., lacquer, is applied onto the can’s internal surface.

As for the failed tuna cans, Barella et al. found superficial welding irregularities such that adhesion of the polymer coating was compromised. Usually, that would not constitute a problem because the oil provides a good protective environment against oxidation. Unfortunately, in this batch of cans oil with high water content (double than normal) was used, thus making the environment more corrosive and eventually leading to the contamination problems that emerged a few months after production.

**Fig. 5:**
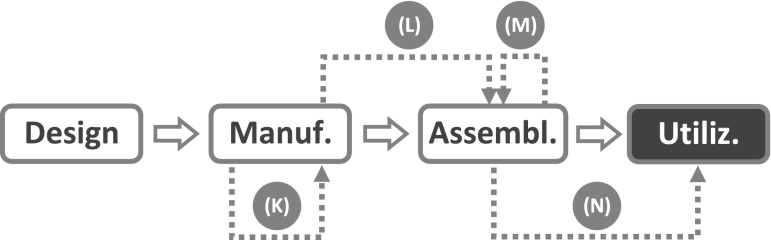
Life cycle model of the failure of tuna cans described in (Barella et al. 2011)

To summarize, two conditions occurred: inadequate welding and oil with high water content. Each condition alone would be unable to cause the failure, but their co-occurrence determined the adverse outcome. This failure scenario can be represented by a life cycle model like the one in Fig. 5 where the production of the tuna cans has been split into two stages: manufacturing, in which the steel sheets are transformed into lacquered cans; and assembly, in which tuna and oil are poured into the cans.

The failure trajectory starts out with the inadequate welding during manufacturing, arrow (K). Since, for unspecified reasons, the quality checks were ineffectual, the defective cans progressed to the assembly stage, arrow (L), where oil with unusually high water content was used to fill them, arrow (M). The joint effect of (L) and (M) is represented by the final step of the failure trajectory leading to product failure during the utilization stage (N).

In Fig. 5, the utilization stage is represented by a shaded rectangle to underline the fact that it is where the product failure is situated. Sure enough, the tuna cans failed to achieve the goal of delivering edible food to the end-users. However, the engineering and business team in charge of the project may ponder that still other goals have been missed such that the mistakes and shortcomings during manufacturing and assembly might be considered failures in their own right. Especially the problem of the defectively welded cans is likely to be taken as an instance of failure during manufacturing, which might be addressed by means of corrective measures like better procedures, improved training, and so on.

## Conclusion

In this paper, the traditional notion of failure has been identified as the termination of the ability of an item to perform a required function. It has been shown, however, that engineers use the notion of failure in a broader sense. Examples from the engineering literature have been given that violate three of the four assumptions on which the traditional approach is based. These assumptions, and the emphasis given to the functions required by end-users, have been analyzed as due to the traditional approach adopting the sequential model of product development as part of its conceptual background. In this model, the end-user requirements are established early on in the development process and determine the framework within which the needs of other stakeholders are addressed in the following steps.

Actual engineering practice is progressively moving away from this sequential model and switching to integrated models in which the notion of product life cycle plays a prominent role. Inspired by the life cycle approach, a new definition for failure has been proposed, *product failure*, and it has been shown that, besides being able to deal with the traditional cases of failure, it can deal with the problematic cases as well. In conjunction with the new definition, the notion of *failure trajectory* has been introduced and it has been argued that the life cycle approach enables the introduction of new trajectories in addition to the three envisioned by the traditional approach.

In these alternative models, product development is led by a trans-disciplinary team that conveys the views of the multiple stakeholders involved in the different stages of the life cycle. As a consequence, a life cycle approach to failure abandons the priority granted to the end-user and switches to a broader view that accounts for the needs of a multiplicity of stakeholders. The new approach can naturally account for failures occurring during the manufacturing stage when a product has not yet started to perform its required functions.

Especially interesting is the possibility of analyzing failure trajectories that point to stages in the life cycle occurring after utilization. The performance of products during these stages is becoming increasingly important because of the impact on sustainability. The retirement stage of a product can be almost as complex as the manufacturing stage and, from the point of view of many stakeholders, no less relevant. Also with respect to retirement a product can be a success or a failure. The life cycle approach makes the notion of failure ready for the sustainability challenges of the twenty-first century.

## Footnotes

1 There are few exceptions to this general attitude. One exception is to maintain that some failures, like near misses, are not *totally* negative because they provide valuable learning opportunities that can be exploited for preventing more serious consequences in the future. Another exception is the approach of material scientists, some of whom maintain that failures are but physical events and processes that have measurable effects on the structure of material samples, e.g., fracture of a material occurs when the tensile stress overcomes the load bearing capacity (Dasgupta and Pecht 1991). From this perspective, failures are not more negative than oxidative processes, fires, are for chemists.
